# Prevalence and clinical relevance of liver dysfunction after thoracic surgery: a retrospective study

**DOI:** 10.1038/s41598-023-49427-0

**Published:** 2023-12-27

**Authors:** Rosanna Villani, Domenico Loizzi, Antonia Federica Sacco, Lucia Mirabella, Mariateresa Santoliquido, Diletta Mongiello, Francesco Sollitto, Gaetano Serviddio

**Affiliations:** 1 C.U.R.E. (University Center for Liver Disease Research and Treatment), Liver Unit, Department of Medical and Surgical Sciences, University of Foggia, Viale Pinto 1, 71122 Foggia, Italy; 2 Institute of Thoracic Surgery, Department of Medical and Surgical Sciences, University of Foggia, Foggia, Italy; 3 Anesthesia and Intensive Care Unit, Department of Medical and Surgical Sciences, University of Foggia, Foggia, Italy

**Keywords:** Hepatology, Liver diseases

## Abstract

Postoperative elevation of serum aminotransferase or alkaline phosphatase levels after liver and heart surgeries has been widely reported. The prevalence and clinical significance of hypertransaminasemia/liver dysfunction after thoracic surgery remains largely unknown. Significant differences in surgical procedures between thoracic and extra-thoracic surgeries may suggest different risks of liver dysfunction. We retrospectively analyzed data from 224 consecutive patients who underwent thoracic surgery. Liver function tests were recorded the day before surgery, 12 h, 1 day, 5, and 10 days after the surgical procedure. Patients were studied to identify the frequency of hypertransaminasemia and/or hyperbilirubinemia and/or increase of INR levels. 37,5% of patients showed an increase in serum alanine aminotransferase (ALT) level after thoracic surgery, whereas an increase in gamma glutamyl transferase (GGT) serum levels of any grade was observed in 53,6% of patients. Approximately 83% of patients who experienced an increase in the serum GGT or ALT levels showed a grade 1 or 2 change. Operative time was associated with hypertransaminasemia in the univariate and multivariate analyses, whereas the use of metformin was associated with a lower risk of ALT increase.

## Introduction

Post-operative alterations in liver function tests have been reported after liver and cardiac surgery, with a prevalence between 25 and 75%^1,2^.

After these procedures, isolated increases in alanine aminotransferase (ALT) levels, hyperbilirubinemia, alteration of the coagulation profile, or their combination may occur^3,4^.

In patients without pre-existing liver disease who undergo liver or cardiac surgery, elevations in aminotransferases or cholestatic liver enzymes are typically mild, transient, and self-limiting, whereas clinically significant liver dysfunction is more likely to occur in patients with liver disease^1^. More rarely, liver function impairment has been recorded with a more severe clinical presentation characterized by hyperbilirubinemia and increased INR, a clinical scenario defined as postoperative liver dysfunction (POLD), which is associated with a worse clinical outcome or liver failure^5^.

From a pathophysiological perspective, postoperative liver dysfunction may occur for several reasons. Data from the literature suggest that both surgical procedures and anesthesia may contribute to impaired liver function. Anesthesia causes a transient reduction of approximately 35% in hepatic arterial blood flow; therefore, hypoperfusion or reperfusion appears to be involved in the postoperative liver injury^6,7^. In particular, volatile anesthetics seem to be involved in systemic vasodilation and, therefore, in reduction of hepatic arterial blood flow, even if they have a low hepatic metabolism (isoflurane, desflurane, and sevoflurane < 1%). In addition, the surgical procedure may be involved itself, due to direct trauma to the liver or abdominal tissues, in postoperative liver injury. This is why major or liver surgery are associated with a greater risk of hepatic injury than extra-abdominal or minor procedures.

The prevalence, severity, and duration of hypertransaminasemia and liver dysfunction after thoracic surgery remain largely unknown. There were significant differences in anesthetic management and surgical procedures between thoracic and extra-thoracic surgeries, which may suggest the occurrence of distinct clinical scenarios. For example, volatile halogenated anesthetics are currently not recommended over intravenous drugs in thoracic surgery, although they are generally used for anesthesia induction^8–11^.

Therefore, it is reasonable to hypothesize that thoracic surgery has different effects on liver function.

We retrospectively analyzed data from a cohort of patients who underwent thoracic surgery to study the prevalence, clinical features, evolution and risk factors of postoperative liver injury.

## Patients and methods

### Study design and patient population

We conducted a retrospective study and analyzed data from the clinical records of 332 consecutive patients admitted to the Institute of Thoracic Surgery of the Teaching Hospital “Policlinico Riuniti”, University of Foggia, between January 2020 and December 2021.

#### Inclusion and exclusion criteria

All patients undergoing major or minor thoracic surgery during the above-mentioned time range were included in our study.

Major surgery included the following surgical procedures:

1. Lung resection (included atypical resection).
2. Lobectomy.
3. Lung decortication/pleurectomy.

By contrast, minor surgery included:

1. Lung or pleural biopsy.
2. Sympatholysis.
3. Lymphadenectomy.

Exclusion criteria were:

- Age < 18 years,
- Chronic viral hepatitis,
- Liver cirrhosis,
- Autoimmune liver disease,
- Increase in ALT, aspartate aminotransferase (AST), and gamma-glutamyl transferase (GGT) (any grade) at baseline.
- Use of anticoagulant drugs.

### Liver function biochemical profile

Hemoglobin (Hb), hematocrit, platelet count, white blood cell count, alanine aminotransferase (ALT), aspartate aminotransferase (AST), gamma-glutamyl transferase (GGT), alkaline phosphatase (ALP), albumin, total bilirubin, direct bilirubin, and serum creatinine levels.

Blood examinations were performed at multiple time points according to the protocol of the Institute of Surgery.

- Baseline: the day before surgery (day − 1);
- 12 h after surgery (day 0);
- 24 h after surgery (day 1);
- 5 days (day 5);
- 10 days (day 10).

The severity of AST, ALT and GGT increase was classified as follows:

- Up to 1.5 Upper Limit of Normal (ULN) (grade 1);
- 1.5–3 (ULN) (grade 2);
- 3–5 ULN (grade 3);
- 5–20 ULN (grade 4);
-  > 20 ULN (grade 5).

We studied our population to identify one of the following clinically significant scenarios:

- **Post-Operative hyperTransaminasemia** (**POT**): increased ALT levels of any grade in patients with normal ALT levels before thoracic surgery.
- **Post-Operative hyperBilirubinemia (POB)** is defined as hyperbilirubinemia of any grade in patients with normal bilirubin levels before thoracic surgery.
- **Post-Operative increase in INR level** (**POI**): defined as INR > 1.2 or the need for clotting factors to maintain normal INR in patients with normal values at baseline.
- **COmbined Post-operative Alterations of Liver function tests** (**COPAL**): when two of the previous biochemical alterations were observed (POT + POB or POT + POI or POB + POI).
- **Post-operative Liver Dysfunction** (**POLD**).

Post-operative liver dysfunction was defined by the following criteria.

- Increase in ALT levels (any grade) and
- Increased INR (or need for clotting factors to maintain a normal INR) and
- Hyperbilirubinemia (any grade) and
- Exclusion of biliary obstruction.

The upper limits of normal values was 1.2 for INR and 1.2 mg/dl for total bilirubin.

The study was approved by the Ethics Committee of the Teaching Hospital “Policlinico Riuniti” of Foggia and was conducted according to the ethical standards of our institutional research committee and the 1964 Declaration of Helsinki and its later amendments. All participants provided written informed consent to the study.

## Anesthetic protocol

Patients scheduled to undergo major surgery required one lung ventilation (OLV) in the right or left lateral position. Therefore, upon their arrival in the operating room, an epidural catheter was placed in T5–T6 in mid approach. Epidural infusion 0.25% ropivacaine with fentanyl 2 µg/ml was used at a rate adjusted to patient’s height, 5 ml/h up to 160 cm, 0.5 ml increment for every 5 cm > 160 cm. Patients were pre-medicated with midazolam 0.03–0.04 mg/kg. After applying standard monitoring device, radial artery was cannulated and the cannula was connected to the FloTrac™ sensor and the Vigileo™ monitor (Edwards Life Sciences LLC, Irwine, CA, USA, software version 01.10), which, in addition to arterial pressure transduction, allowed stroke volume (SV) and stroke volume variation (SVV) estimation from the arterial pressure waveform. The level of anesthesia was assessed through bi-spectral index (BIS) monitoring (Aspect A-2000^®^; Aspect Medical System, Newton, MA). Patients were given a bolus of 8 ml/kg of normal saline IV before the induction of anesthesia and were maintained with 5 ml/ kg/h of normal saline solution. Anesthesia was induced with propofol 2 mg*kg^−1^, fentanyl 3 γ*kg^−1^, and succinylcholine 1 mg*kg^−1^. Anesthesia was maintained with an infusion of propofol 150–200 γ*kg^−1^*min^−1^, remifentanil 0.1–0.2 γ*kg^−1^*min^−1^ and cisatracurium 1.5 γ*kg^−1^*min^−1^. The infusion rate of propofol was adjusted in order to target a bispectral index (BIS) value between 50 and 60. In minor surgery procedures, paravertebral block was placed at the level of the surgical thoracic incision and 0.2% of levobupivacaine was used. No patients need inotropic drugs during surgery.

### Statistical analysis

Quantitative variables are presented as median and interquartile range (IQR). Continuous variables were compared using the Mann–Whitney U test and categorical variables were compared using Fisher's exact test. Univariate and multivariate logistic regression analyses were used to identify associations between clinical parameters and the risk of new-onset liver hypertransaminasemia/hyperbilirubinemia/POLD. Statistical significance was set at p value < 0.05.

The diagnostic value of each variable and combination of multiple variables were assessed using the area under the receiver operating characteristic (ROC) curve. Different curves were compared by using the DeLong test^12^. Statistical analyses were performed using SPSS (Statistical Package for the Social Sciences, version 20; Armonk, New York, NY, USA) and GraphPad Prism version 9 (La Jolla, CA, USA).

## Results

### Baseline characteristic of study population

Study population included 332 patients.

108 patients were excluded because:

- 64 patients had an increase of any grade in ALT or GGT or bilirubin level at baseline,
- 12 patients had a previous diagnosis of chronic hepatitis or liver cirrhosis,
- 32 patients were taking anticoagulant.

Table 1 shows the baseline characteristics of the study population included in the final analysis (N = 224).

**Table 1:** Baseline characteristics of study population.

Variable	Study population N = 224	Patients with increased ALT N = 84 (37.5%)	Patients with normal ALT N = 140 (62.5%)	p
Age, years	65 (51–74)	62 (51–70)	61 (51–74)	n.s
Male gender, %	143 (63.8%)	55 (65.5%)	94 (63.9%)	n.s
Length of hospitalization, days	9 (7–12)	9 (6–12)	9 (7–12)	n.s
Operative time*				
Major surgery (min)	100 (70–140)	110 (75–125)	95 (70–140)	n.s
Minor surgery (min)	55 (35–75)	60 (40–75)	55 (35–70)	
Major surgery, N (%)	146 (65.2%)	49 (58.3%)	97 (69.3%)	n.s
Lung cancer, N (%)	177 (79%)	61 (79.2%)	116 (78.9%)	n.s
Diabetes, N (%)	38 (17%)	6 (7.8%)	32 (21.8%)	**0.002**
Arterial hypertension, N (%)	99 (44.2%)	31 (40.3%)	68 (46.3%)	n.s
Heart failure, N (%)	65 (29%)	19 (24.7%)	46 (31.3%)	n.s
Kidney failure (all grades), N (%)	94 (41.9%)	42 (50%)	52 (37.1%)	n.s

Significant values are in bold.

*Expressed as medians and interquartile ranges.

The number of patients with diabetes was significantly different between the subgroups (Table 1); therefore, we also recorded the data on antidiabetic drugs. 24 out of 38 patients were taking one or more oral antidiabetic drugs, and all of them were taking metformin (maximum daily dose between 500 and 2000 mg). Twelve patients were treated with insulin according to the basal-bolus (N = 8 patients) or basal regimen (N = 4 patients). Three of the four patients treated with the basal regimen also received metformin (maximum daily dose between 500 and 2000 mg).

### Overall prevalence of Post-operative hyperTransaminasemia (POT) after thoracic surgery and serum GGT levels

In our study population 37.5% of patients showed an overall increase in serum ALT levels after thoracic surgery, whereas increase in AST levels occurred in 30.3% of patients. An increase in GGT serum levels of any grade was observed in 53.6% of patients.

**Figure 1:**
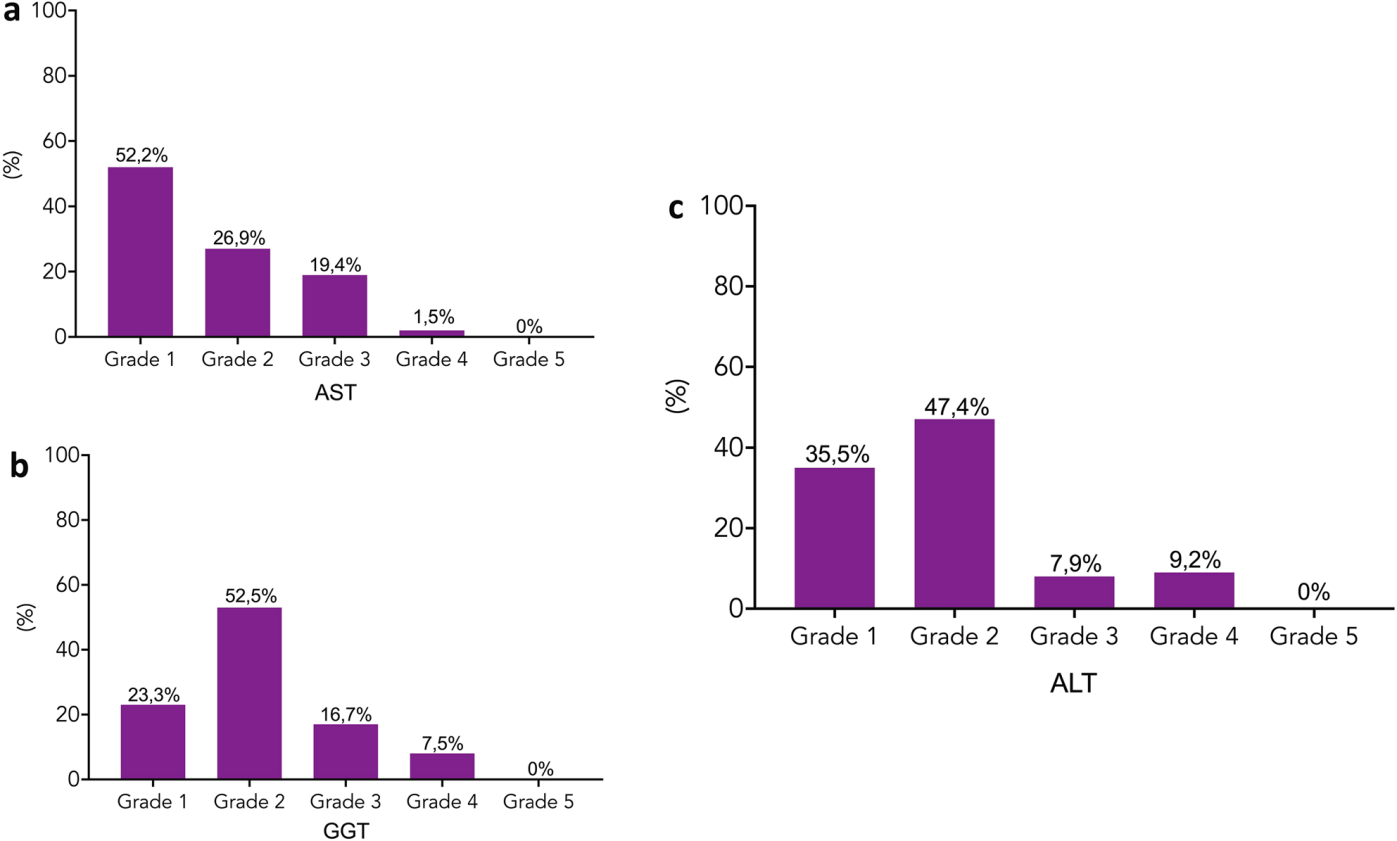
Frequency of alteration of AST (**a**), GGT (**b**) and ALT (**c**) by severity in our study population. Grade 1: Up to 1,5 ULN; grade 2: 1,5–3 ULN; grade 3: 3–5 ULN; grade 4: 5–20 ULN; Grade 5 > 20 ULN.

The prevalence of liver function alterations according to the severity subgroup is shown in Fig. 1. Approximately 50% of patients who experienced an increase in the serum GGT or ALT levels showed a grade 2 level change. Approximately 83% of patients who experienced an increase in the serum ALT levels showed a grade 1 or 2 level change. Similarly, approximately 76% of patients with increased GGT levels showed a grade 1 or 2 level change.

No cases of liver failure or grade 5 (severe acute hepatitis) were recorded in our cohort.

**Figure 2:**
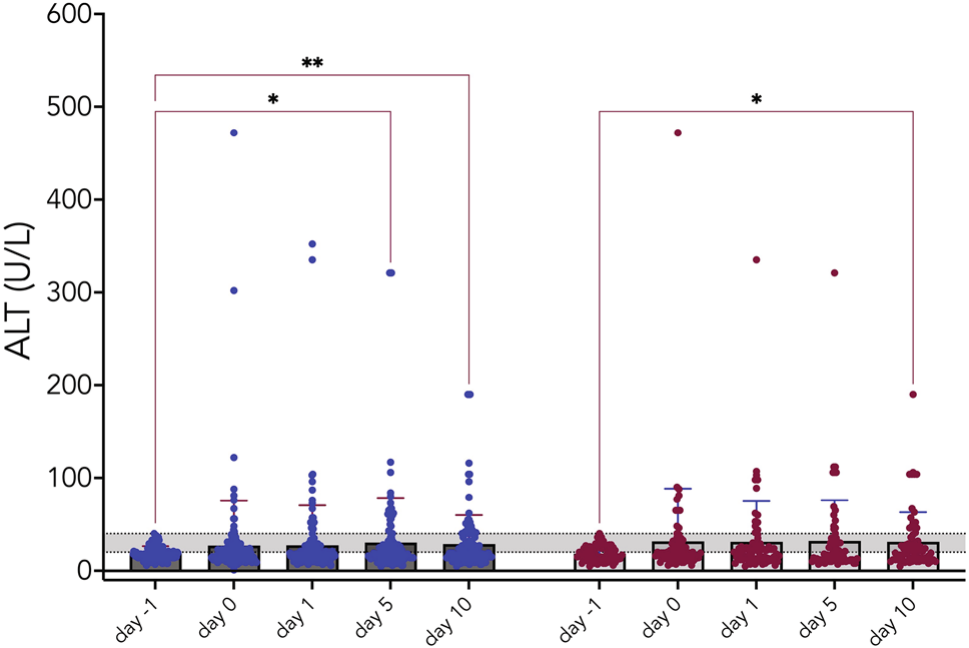
Temporal trend of serum ALT levels in patients who underwent major (blue) or minor (red) thoracic surgery.

Figure 2 and Supplementary Fig. 1 show the temporal trends of ALT and GGT levels after thoracic surgery in the surgical procedure subgroup (major versus minor surgery).

Major surgery was associated with a significant increase in ALT levels at days 5 and 10 after the surgical procedure, whereas minor surgery was associated with a borderline significance at day 10 after surgery. Supplementary Fig. 1 shows a significant increase in GGT levels on day 10, irrespective of the surgical procedure.

### Risk factors for Post-operative hyperTransaminasemia (POT) and increased serum GGT levels after thoracic surgery

Tables 2 and Supplementary Table 1 shows the results of the univariate and multivariate analyses exploring the potential risk factors associated with serum ALT and GGT alterations. For hypertransaminasemia, significant results were recorded for diabetes and major surgery, whereas for increased GGT levels, only major surgery was significant.

**Table 2:** Variables associated with increased ALT level during the hospital stay in patients undergoing thoracic surgery.

Variable	Univariate analysis			Multivariate analysis		
	B	OR (95%CI)	p-value	B	OR (95%CI)	p-value
Gender, male	0.114	1.121 (0.637–1.073)	0.693			
Age, years	− 0.016	0.985 (0.968–1.002)	0.078			
SCC, yes	0.223	1.249 (0.682–2.287)	0.471			
Operative time	− 0.005	0.994 (0.984–0.995)	**0.039**	− 0.005	0.995 (0.989–1.000)	**0.044**
albumin	− 0.174	0.840 (0.531–1.329)	0.457			
Pseudocholinesterase	0.000	1.000 (1.000–1.000)	0.566			
eGFR	− 0.004	0.996 (0.990–1.003)	0.259			
Fibrinogen	0.002	1.003 (1.002–1.005)	**0.047**			
Hb	− 0.084	0.919 (0.808–1.046)	0.201			
PLT	0.000	1.000 (1.000–1.000)	0.564			
Diabetes, yes	− 1.349	0.260 (0.104–0.651)	**0.004**	− 1.477	0.228 (0.089–0.587)	**0.002**
Metformin use, yes	− 1.087	0.337 (0.133–0.858)	**0.022**	− 1.164	0.312 (0.120–0.813)	**0.017**
Major surgery	− 0.477	0.621 (0.353–1.090)	0.097			
PT	− 0.028	0.972 (0.952–0.993)	**0.010**	− 0.026	0.974 (0.952–0.998)	**0.030**

Significant values are in bold.

The significance of association was assessed by univariate and multivariate logistic regression analysis.

Because of the statistical significance for the variable “diabetes” and, due to the high frequency of metformin use in the diabetic subgroup (71%), we performed a univariate and multivariate analysis including the use of metformin as variable and we found a OR = 0.337 (0.133–0.858; p = 0.022) in univariate analysis and a OR = 0.312 (0.120–0.813; p = 0.017) in multivariate analysis. Age and sex were not significantly associated with liver dysfunction in the univariate and multivariate analyses.

### Post-Operative hyperBilirubinemia (POB)

Overall, hyperbilirubinemia occurred in sixty-four patients (28.5%). Among them, 46.9% had unconjugated, 4.7% conjugated and 48.4% mixed hyperbilirubinemia. Among patients with hyperbilirubinemia, twenty-nine patients (12.9%) showed an isolated increase in total bilirubin over the upper limit of normality without changes in INR or ALT values,whereas 15.6% of patients experienced hyperbilirubinemia associated with hypertransaminasemia (7.1%) or increased INR (8.5%). Among patient who experienced an increase of any grade in serum bilirubin levels, only one patients reached a total bilirubin > 3 mg/dl.

### Risk factors for hyperbilirubinemia after thoracic surgery

Table 3 shows the results of univariate and multivariate analyses for the appearance of hyperbilirubinemia. The Hb levels have shown a significant correlation with the occurrence of hyperbilirubinemia (OR: 1.247; 95% CI 1.045–1.490; p = 0.015), whereas sex, age, operative time, and type of surgery were not statistically significant*.*

**Table 3:** Variables associated with hyperbilirubinemia during the hospital stay in patients undergoing thoracic surgery.

Variable	Univariate analysis			Multivariate analysis		
	B	OR (95% CI)	p-value	B	OR (95% CI)	p-value
Gender, female	− 0.611	0.543 (0.287–1.027)	0.061			
Age, years	− 0.007	0.993 (0.975–1.011)	0.542			
SCC, yes	0.636	1.889 (0.945–3.777)	0.072			
Operative time	0.001	1.001 (0.997–1.006)	0.547			
Albumin	0.549	1.731 (1.042–2.875)	**0.034**			
Pseudocholinesterase	0.000	1.000 (1.000–1.000)	0.112			
eGFR	− 0.002	1.000 (0.994–1.004)	0.962			
Fibrinogen	− 0.002	0.998 (0.997–1.000)	0.094			
Hb	0.248	1.281 (1.076–1.526)	**0.005**	0.221	1.247 (1.045–1.490)	**0.015**
PLT	0.000	1.000 (1.000–1.000)	0.567			
Metformin use, yes	− 0.173	0.841 (0.395–1.789)	0.653			
Major surgery	− 0.325	0.723 (0.387–1.350)	0.309			
PT	0.010	1.010 (0.987–1.034)	0.386			

Significant values are in bold.

The significance of association was assessed by univariate and multivariate logistic regression analysis.

### POI value after thoracic surgery

22 patients (9.8%) showed an isolated increase in INR value after the surgical procedure, all of them had diabetes and/or cardiovascular disease. In these patients, only mild changes in INR values occurred and the highest INR value was recorded in a 74-year-old diabetic female who underwent major surgery for lung cancer (INR, 1.58).

### Post-operative liver dysfunction

We observed only four cases of POLD in our patient cohort. Supplementary Table 2 shows the biochemical and clinical features of these patients. All the patients with POLD had normal hepatic cytolysis and cholestasis before surgery. All cases did not showed a life-threatening clinical course but only a mild form of liver dysfunction.

### Predictive models for the identification of patients with post-operative hypertransaminasemia

Variables associated with the diagnosis of postoperative hypertransaminasemia were first assessed using univariate analysis and then studied using the area under the curve (AUC).

Operative time (AUC 0.57; 95% IC 0.49–0.65), fibrinogen (AUC 0.59; 95% IC 0.51–0.67) and PT (AUC 0.40; 95% IC 0.32–0.47) were not discriminators for the diagnosis of hypertransaminasemia during hospitalization when used alone.

Therefore, using stepwise logistic regression, we tested the combined predictors of the development of hypertransaminasemia.

**Figure 3:**
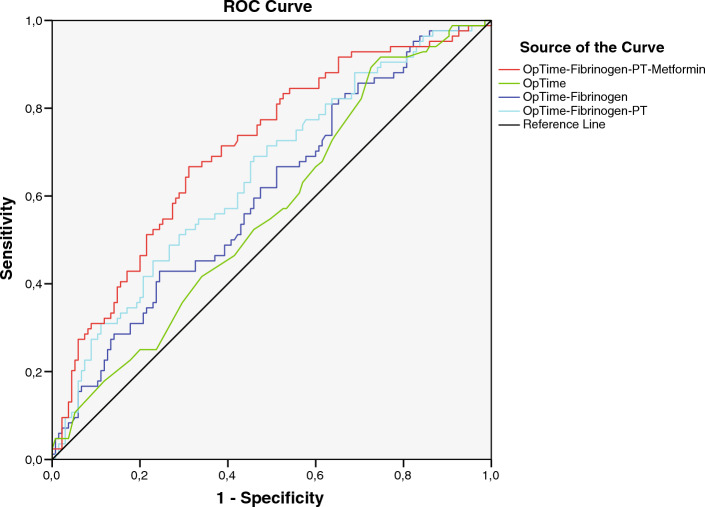
AUROC curve including operative time, fibrinogen, PT, and metformin use. When all variables are included in analysis AUROC is 0.71 (95% IC 0.64–0.78).

The optimal combination included operative time, fibrinogen level, PT, and metformin use. As shown in Fig. 3, together they reached an area under the ROC curve of 0.71 (95% IC 0.64–0.78). Stepwise logistic regression with backward and forward methods yielded similar findings.

The combination of operative time, fibrinogen and PT reached an AUC of 0.65 (95% IC 0.57–0.72) whereas operative time and fibrinogen were associated with a lower AUC (0.61; 95% IC 0.53–0.68).

Comparing the AUCs using the DeLong test, we found that the AUC obtained from the combination of operative time, fibrinogen, PT, and metformin use was statistically different from all other curves (p < 0.001 versus AUC from operative time; p = 0.0014 versus AUC obtained from the combination of operative time and fibrinogen; p = 0.0093 versus AUC obtained from the combination of operative time, fibrinogen, and PT).

Similarly, the AUC obtained from the combination of operative time, fibrinogen level, and PT was significantly different when compared with the AUC from operative time (p = 0.0006) but not different from the AUC from operative time and fibrinogen level (p = 0.08).

The optimal threshold values for the continuous variables used to calculate the AUCs are as follows:

- Operative time, 130 min (sensitivity 91.7%, specificity 25.7%; VPP 55.4%; VPN 77.4%)
- Fibrinogen 400 mg/dl (sensitivity 48.8%; specificity 71.3%; VPP 62.8%; VPN 58.2%)
- PT 89% (sensitivity, 50.6%; specificity, 65.3%; VPP 59,3%; VPN 57.1%)

## Discussion

Hypertransaminasemia, or transient hyperbilirubinemia, is a frequent finding in patients undergoing liver and heart surgery^13^. A large body of literature is available on the association between liver surgery or anesthesia and transient liver injury, which may sometimes evolve into global liver dysfunction, the so-called postoperative liver dysfunction^14^.

Liver surgery has an intrinsic risk of liver dysfunction and mortality in approximately 5% of patients, depending on the extent of resection and the quality of the liver parenchyma^15^. Similarly, in cardiac surgery, approximately 10% of patients who undergo cardiopulmonary bypass experience hepatic injury, which directly affects morbidity and mortality of these patients^16,17^. In this case, several pathophysiological mechanisms, such as systemic inflammatory response syndrome, centrilobular ischemia with subsequent reperfusion injury, drug-induced injury, or consumption of coagulation factors with microthrombus formation, may be involved in the mechanisms that lead to hepatic cell injury^16,18,19^.

No data are available for patients who underwent thoracic surgery, which is significantly different from abdominal and cardiac surgeries because of different surgical techniques and anesthesiology management.

Therefore, for the first time, we analyzed the prevalence and clinical impact of thoracic surgery on liver function. Moreover, we studied the risk factors for the early identification of patients who may be at a high risk of developing more severe liver dysfunction or may deserve close biochemical follow-up to optimize postoperative management.

In our study 37.5% of patients showed an increase in serum ALT levels (any grade), whereas 53.6% of patients showed an increase in GGT serum levels of any grade.

83% of patients who experienced an increase in ALT levels showed a grade 1 or 2 alteration, whereas approximately 17% had a grade 3 or 4 change. In most cases, the hypertransaminasemia was mild and lasted no longer than 10 days.

Similarly, among patients who experienced an increase in serum GGT levels, approximately 75% of them showed a grade 1 or 2 change, in other words, a mild liver impairment.

We did not record cases of liver failure or grade 5 increases in ALT and GGT levels, suggesting that the risk of severe liver injury may be unusual in patients without liver disease.

Only 4 cases of POLD were recorded; however, hyperbilirubinemia was not higher than 3.4 mg/dl suggesting that only a mild form of POLD was observed in our cohort.

Hyperbilirubinemia occurred in 28,6% of patients and 12,9% of patients had an isolated value of total bilirubin > 1.2 mg/dl of any grade, whereas only 1 patient had total bilirubin > 3 mg/dl.. Among patients with hyperbilirubinemia, most of them had unconjugated (46,9%) or mixed (48,4%) hyperbilirubinemia. Unconjugated hyperbilirubinemia can be recorded in patients with blood transfusions, hematomas or because of drug effects; all these conditions may be observed in patients underlying thoracic surgery and they don’t inevitably suggest the presence of liver dysfunction but rather a transient increased catabolic degradation of hemoglobin or other heme protein. Normally, patients with normal liver function efficiently conjugate and excrete the excess bilirubin and, therefore, the increase in serum unconjugated bilirubin is modest and rarely exceed 4 mg/dL.

Similarly, unconjugated hyperbilirubinemia may be caused by drugs interfering with the bilirubin uptake or conjugation such as antibiotics or drugs used for the management of pain during the post-operative phase which inhibit the UGT1A1 enzyme.

**Figure 4:**
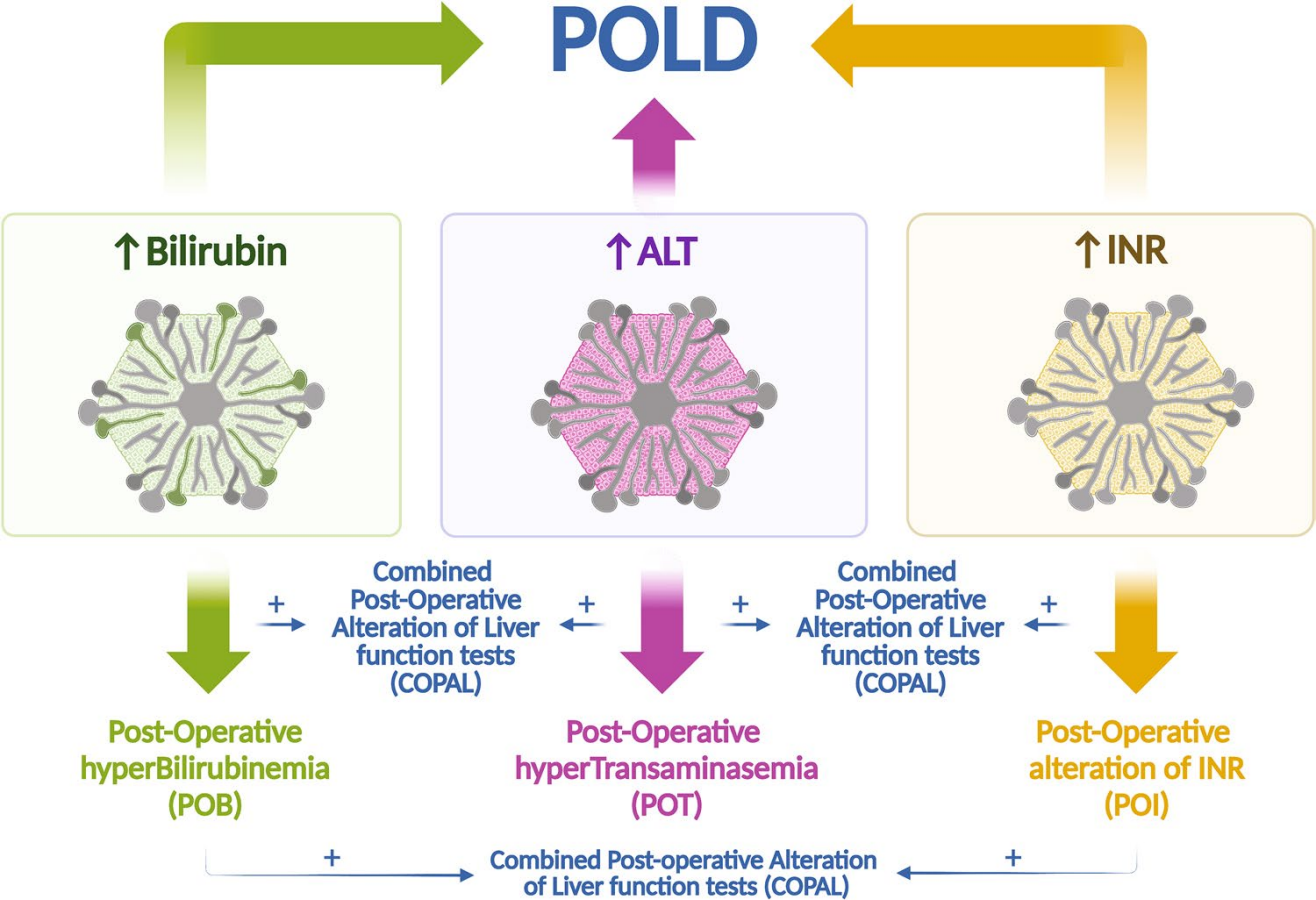
Clinical potential scenarios after thoracic surgery. Post-operative liver dysfunction (POLD) is defined by the concurrent occurrence of hypertransaminasemia (POT), hyperbilirubinemia (POB) and alterations of INR values (POI) when biliary obstruction is excluded. However, in most cases patients do not have criteria for POLD but may have one or more of these alterations. *POT* post-operative hypertransaminasemia, *POB* post-operative hyperbilirubinemia, *POI* post-operative increase in INR level. The combination of 2 alteration is defined COPAL (combined post-operative alterations of liver function tests).

Figure 4 summarize the potential scenarios the surgeons and clinicians may find during the post-operative management of patients who undergo thoracic surgery.

Overall, our data show that hypertransaminasemia is common in patients who undergo thoracic surgery, and that its dysfunction is generally mild. Our data excluded a potential risk for progression to life-threatening liver injury in patients without underlying liver diseases because we observed a transient and benign course, even when the alteration of liver function tests seemed to be severe in the early postoperative phase.

These data are useful to understand the need for additional clinical support from hepatologists following major surgical procedures.

Our data suggest that even grades 3 and 4 hypertransaminasemia do not seem to be associated with short- or long-term complications. Some patients may require intensive follow-up during the first few days after surgery, owing to the potential risk of liver failure. For this purpose, we explored the potential usefulness of predictive models for identifying patients who have a higher risk of developing hypertransaminasemia and, therefore, a higher risk of liver dysfunction after thoracic surgery. In our model, an operative time longer than 130 min, fibrinogen level > 400 mg/dl as a marker of systemic inflammation, alteration of PT value, and the non-use of metformin seemed to be the best predictors of liver dysfunction.

Interestingly, we found that patients taking metformin had a lower risk of developing liver dysfunction. The potential beneficial role of metformin in patients undergoing surgical procedures has been previously explored even in different surgical settings. Diabetic patients taking metformin showed a lower risk of postoperative septicemia, acute renal failure, and 30-day mortality compared with patients who did not use metformin, in both sexes and in every age group in a large cohort of patients (N = 91 356) undergoing major surgery^20^. Similarly, Reitz et al. reported the results of a multicenter study (15 community and academic hospitals) including 10 088 individuals with diabetes who underwent major surgical intervention and found that metformin use was associated with a reduced risk of 90-day mortality and readmission. The study population included patients who underwent major surgery and general, cardiothoracic, neurologic, orthopedic, vascular, gynecological, or urologic surgery^21^.

The role of metformin, beyond its antidiabetic effects, has been widely discussed in medical literature, and data have shown an interesting protective action with a not completely elucidated mechanism. Foretz et al. reported that the protective action of metformin could be due to the inhibition of the opening of the mitochondrial permeability transition pore, which is involved in cellular apoptosis^22^.

The results of our analysis have interesting implications for the clinical practice because surgeons generally refer to hepatologists for the management of liver alterations of all types and grades.

However, taking into account our results, the real need for an hepatological support seems to be restricted to very few patients who may be selected according to specific aspects such as operative time, systemic inflammation, alteration of marker of liver synthesis and the use of drugs which may have a protective effect of hepatocytes such as metformin.

In our study population, we intentionally excluded patients with liver disease to understand the role of thoracic surgery per se in the development of liver injury after minor or major surgeries.

Additional studies are required to address the risk of liver dysfunction in patients with liver disease.

### Supplementary Information

Supplementary Information.

## Data Availability

All data generated or analyzed during this study are included in this published article.
