# Supplementary material for: Prevalence and clinical relevance of liver dysfunction after thoracic surgery: a retrospective study
Source: Sci Rep. 2023 Dec 27;13:23045. doi: 10.1038/s41598-023-49427-0 (PMC10754851; doi:10.1038/s41598-023-49427-0)
Supplement: Supplementary file 1 — Supplementary Information. [file 41598_2023_49427_MOESM1_ESM.docx]

**PREVALENCE AND CLINICAL RELEVANCE OF LIVER DYSFUNCTION AFTER THORACIC SURGERY: A RETROSPECTIVE ANALYSIS**

Rosanna Villani ^*#1^, Domenico Loizzi ^#2^, Antonia Federica Sacco^1^, Lucia Mirabella^3^, Mariateresa Santoliquido^1^, Diletta Mongiello^2^, Francesco Sollitto^2^, Gaetano Serviddio^1^

^1^ C.U.R.E. (University Center for Liver Disease Research and Treatment), Liver Unit, Department of Medical and Surgical Sciences, University of Foggia, Foggia, Italy

^2^ Institute of Thoracic Surgery, Department of Medical and Surgical Sciences, University of Foggia, Foggia, Italy

*** Corresponding author**: Rosanna Villani, C.U.R.E. (University Center for Liver Disease Research and Treatment), Liver Unit, Department of Medical and Surgical Sciences, University of Foggia, Viale Pinto 1, 71122 Foggia, Italy. E-mail: [rosanna.villani@unifg.it](mailto:rosanna.villani@unifg.it).

Phone: +39 0881 733636

**SUPPLEMENTAL DATA**

**SUPPLEMENTAL Table 1: Risk factors for altered GGT levels.**

| Variable | Univariate analysis | | | Multivariate analysis | | |
| --- | --- | --- | --- | --- | --- | --- |
|  | **B** | **OR (95%CI)** | **p-value** | **B** | **OR (95%CI)** | **p-value** |
| Gender, female | -0,017 | 0,950  (0,570-1,695) | 0,950 |  |  |  |
| Age, years | -0,005 | 0,995  (0,979-1,012) | 0,594 |  |  |  |
| SCC, yes | 0,094 | 1,098  (0,617-1,956) | 0,750 |  |  |  |
| Operative time | -0,004 | 0,996  (0,992-1,000) | **0,076** |  |  |  |
| Albumin | -0,168 | 0,845  (0,542-1,318) | 0,458 |  |  |  |
| Pseudocholinesterase | 0,000 | 1,000  (1,000-1,000) | 0,165 |  |  |  |
| eGFR | -0,002 | 0,998  (0,992-1,004) | 0,484 |  |  |  |
| Fibrinogen | 0,002 | 1,002  (1,000-1,004) | **0,015** |  |  |  |
| Hb | -0,137 | 0,872  (0,760-1,000) | **0,049** |  |  |  |
| PLT | 0,000 | 1,004  (1,002-1,005) | **0,021** |  |  |  |
| Metformin use, yes | 0,297 | 1,346  (0,668-2,714) | 0,406 |  |  |  |
| Major surgery | 0,828 | 2,288  (1,298-4,031) | **0,004** | 0,682 | 1,978  (1,006-3,888) | **0,048** |
| PT | -0,025 | 0,976  (0,955-0,996) | **0,021** |  |  |  |

**SUPPLEMENTAL Table 2:** Clinical and biochemical features of patients with POLD after thoracic surgery

|  | Age | Gender | Surgery procedure | INR  (Max value) | Bilirubin (mg/dl)  max value | ALT (U/L)  max value | AST (U/L)  max value | GGT (U/L)  max value |
| --- | --- | --- | --- | --- | --- | --- | --- | --- |
| Patient 1 | 70 | M | Major | 1,45  (Day 5) | 3,02  (Day 5) | 46  (Day 5) | 52  (Day 5) | 66  (Day 5) |
| Patient 2 | 76 | M | Major | 1,31  (Day 5) | 3,33  (Day 5) | 321  (Day 5) | 170  (Day 5) | 635  (Day 5) |
| Patient 3 | 64 | M | Major | 1,35  (Day 5) | 1,88  (Day 5) | 67  (Day 5) | 55  (Day 5) | 58  (Day 5) |
| Patient 4 | 21 | M | Major | 1,24  (Day 5) | 3,4  (Day 5) | 67  (Day 10) | 51  (Day 5) | 111  (Day 10) |

**SUPPLEMENTAL FIGURE 1:** Temporal trend of serum GGT levels in patients who underwent major (blue) or minor (red) thoracic surgery.
